# Fast and Sensitive LC-DAD-ESI/MS Method for Analysis of Saikosaponins c, a, and d from the Roots of *Bupleurum Falcatum* (Sandaochaihu)

**DOI:** 10.3390/molecules16021533

**Published:** 2011-02-11

**Authors:** Li-Chun Zhao, Wei Li, Yu-Fang He, Xiao-Hua Li, Zi Wang, Wen-Cong Liu, Yi-Nan Zheng, Jian Liang

**Affiliations:** 1 The Affiliated Ruikang Hospital of Guangxi Traditional Chinese Medical College, Nanning 530011, China; E-Mail: hyzlc@126.com (L.-C.Z.); 2 College of Chinese Medicinal Material, Jilin Agricultural University, Changchun 130118, China; 3 Jilin Academy of Chinese Medicine Sciences, Changchun 130012, China; 4 Department of Crop Science, Chungnam National University, Daejeon 305-754, Korea

**Keywords:** LC-DAD-ESI/MS, saikosaponins, pressurized liquid extraction, *Bupleurum falcatum*

## Abstract

In the present study, we developed a liquid chromatography-diode array detector-electrospray ionization/mass spectrometric (LC-DAD-ESI/MS) method for analysis of saikosaponins in *Bupleurum falcatum*. The LC method employed a ZORBAX SB-Aq analytical column (150 × 4.6 mm i.d., 5 μm) at a flow rate of 0.8 mL/min coupled with a diode array detector at 204 nm. A step gradient of acetonitrile-water (v/v) containing 0.5% formic acid from 30 to 70% was applied, leading to a sample analysis time of 30 min. The ESI-MS was carried out in positive and negative modes from 500 to 1,500 *m/z*. Saikosaponins c, a, and d gave strong sodium adducts at *m/z* 949.6, 803.5 and 803.6, respectively, in positive mode. The data indicate that the present LC-DAD-ESI/MS assay is an effective method for the determination of saikosaponins c, a and d from the roots of *Bupleurum falcatum*.

## 1. Introduction

**Figure 1:**
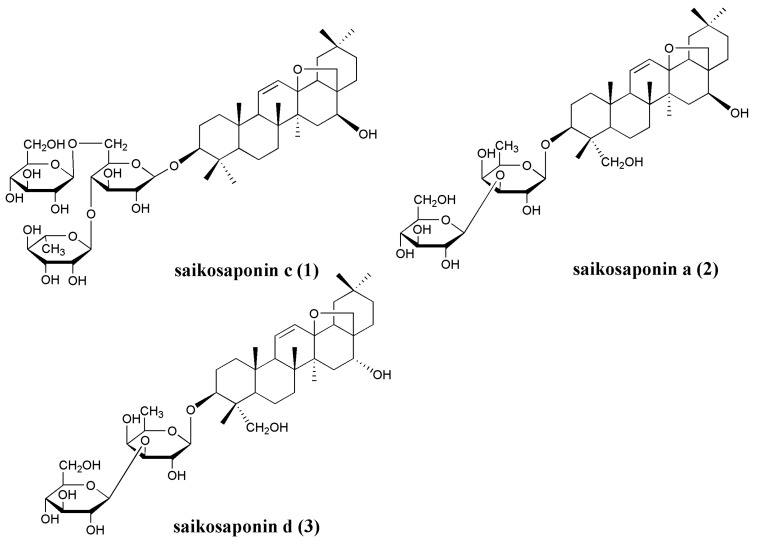
Chemical structures of three major saikosaponins from the roots of *B. falcatum*.

Radix bupleuri (Chaihu in Chinese), as an old member of the family of traditional Chinese herbs, has been used in China to treat cold fevers, chills, and menstrual disorders for more than 2000 years [1]. Generally, Radix bupleuri is derived from the dried roots of *B. Chinese* DC. and *B. scorzonerifolium* Wild. (Umbelliferae). In addition to the above two species, more than ten other ones are available on the Chinese market. Recently, because of the increasing demand as well as a shortage of quality product, *Bupleurum falcatum* (Sandaochaihu in Chinese) as one of the most important cultivars, have been used as medicine in different areas of China. The roots of *B. falcatum* have been used for ailments including influenza [2], renal disease [3,4,5] and mental illness [6,7], *etc*. Several oleanane-saponins, mainly saikosaponins c, a, and d, isolated from *B. falcatum*, are responsible for the broad therapeutic effects identified in its crude drug (Figure 1). Saikosaponins a and d possessed anti-cancer [8,9] and may prevent hepatocyte injury [10], while saikosaponin c showed potential for therapeutic angiogenesis [11].

Concern about of the effect of saikosaponins on human health have raised the issue of using suitable methods for determining their content in *B. falcatum*. To date quite a few approaches have been developed for the determination of these saikosaponins in Radix bupleuri [12,13,14], however, a systematic study on the simultaneous determination of the three saikosaponins from *B. falcatum* by LC-DAD-ESI/MS analysis was not available. In this paper, a fast and sensitive LC-DAD-ESI/MS method for analysis of saikosaponins c, a, and d from the roots of *B. falcatum* was developed, which would be useful for quality control applications to Radix bupleuri and other plants associated with these ingredients.

## 2. Results and Discussion

### 2.1. Optimization of LC Conditions

**Figure 2:**
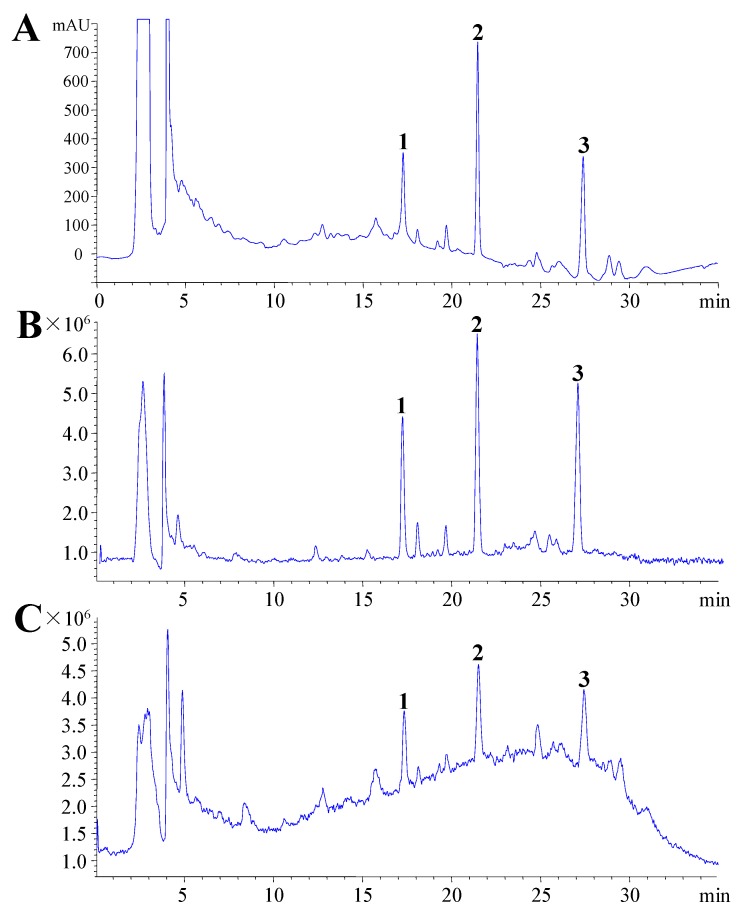
HPLC chromatogram of extracts of *B. falcatum* (A); Total ion chromatogram from *m/z* 500 to 1,500 in negative ion mode (B) and positive mode (C). Peaks: 1. saikosaponin c; 2, saikosaponin a; 3, saikosaponin d.

For LC condition optimization, a Hypersil ODS2 (250 mm × 4.6 mm, i.d., 5.0 μm) and a ZORBAX SB-Aq (150 mm ×4.6 mm, i.d., 5.0 μm) column were tried. There was no appreciable difference in peak shapes and resolution pattern on both columns under similar chromatographic conditions, though it was expected that a longer column might retain these saikosaponins due to their polar nature, therefore, the ZORBAX SB-Aq column was used for further optimization with different mobile phase conditions. Scanning from 200 to 400 and 204 nm was chosen as detection wavelength for acquiring chromatograms [15]. In the present investigation, reducing the acetonitrile content resulted in broadening of peaks without affecting the resolution, so gradient elution with acetonitrile/water at 0.8 mL/min was selected. Under these conditions, saikosaponins c, a and d eluted at 17.27, 21.45 and 27.39 min, respectively, with a run time of 30 min. A chromatogram of a typical sample is shown in Figure 2.

### 2.2. Optimization of Sample Preparation

**Figure 3:**
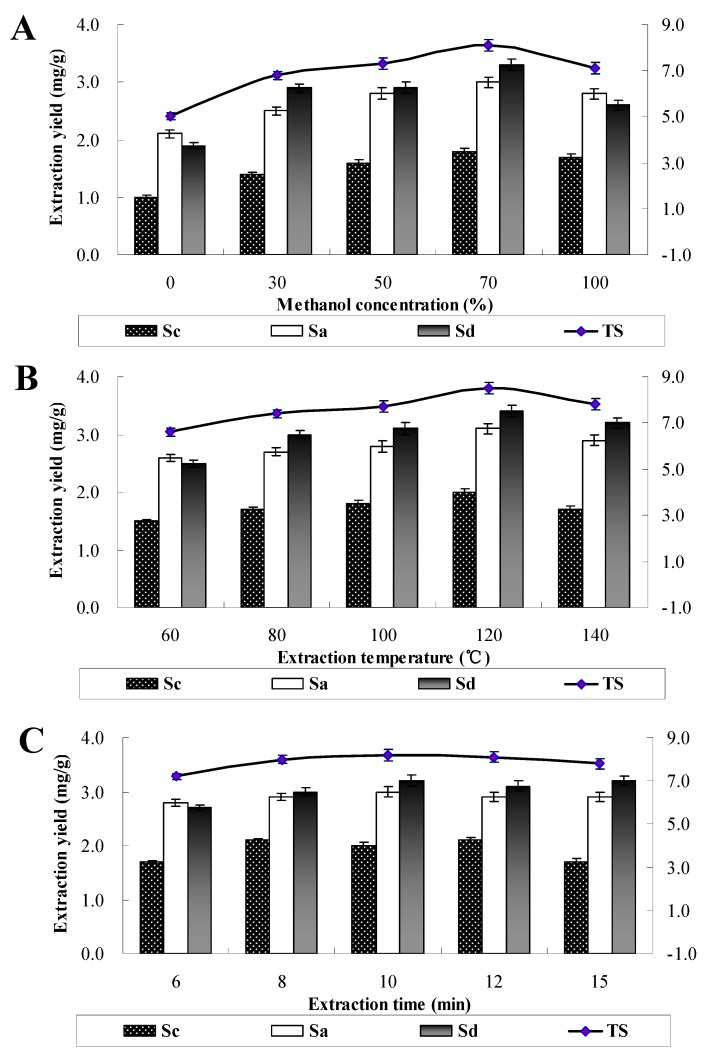
Effects of extraction solvent, temperature and time on yields of three saikosaponins in *B. falcatum*.

Pressurized liquid extraction (PLE) followed by constituents determination has been commonly applied for the analysis of natural compounds in many published papers [16,17]. In order to achieve the optimal extraction conditions, variables involved in the extraction procedure such as extraction solvents, extraction time and extraction temperature were investigated. As shown in Figure 3, under optimal PLE conditions, *i.e.*, 70% methanol as extraction solvent, 120 °C of extraction temperature, 10 min of static extraction time and 60% of flush volume with a single extraction cycle, the yield of total saikosaponins reached a maximum value.

### 2.3. Optimization of LC-MS

Electrospray ionization (ESI) is used for mass spectrometry to produce ions, especially to produce ions from macromolecules because of the propensity of these molecules to fragment when ionized [18]. In the present investigation, the combination of diode array detection (DAD) and electrosapry ionization mass spectrometry, coupled to the LC with a reverse phase column, provided an accurate method for the analysis of saikosaponins in *B. falcatum*. In order to increase the signal-to-noise, 0.5% of formic acid was added to the mobile phase to enhance the molecular ion intensity. Identification of saikosaponins was confirmed by compared it with predominantly [M − H + HCOOH + H_2_O]^−^ and [M − H]^−^ ion in the negative mode and [M + Na]^+^ ion in the positive mode.

At a low ionizing voltage (20 eV) we did not observe the molecular ions, but at 40 eV obvious molecular ions were observable. Hence, the optimized ionizing voltage for saikosaponins was found to be 40 eV.

**Figure 4:**
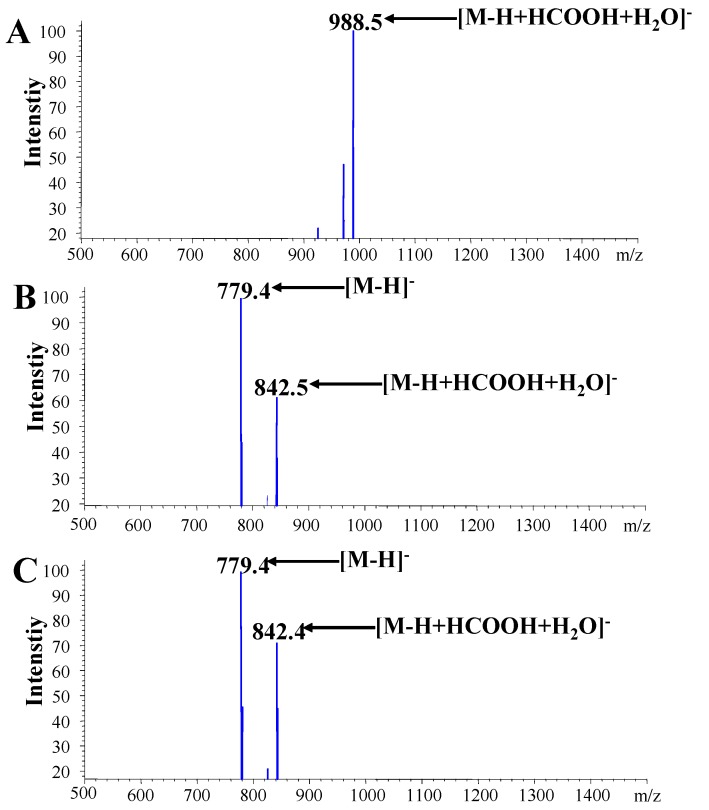
ESI/MS (−) spectra of saikosaponin c (A); saikosaponin a (B); saikosaponin d (C).

**Figure 5:**
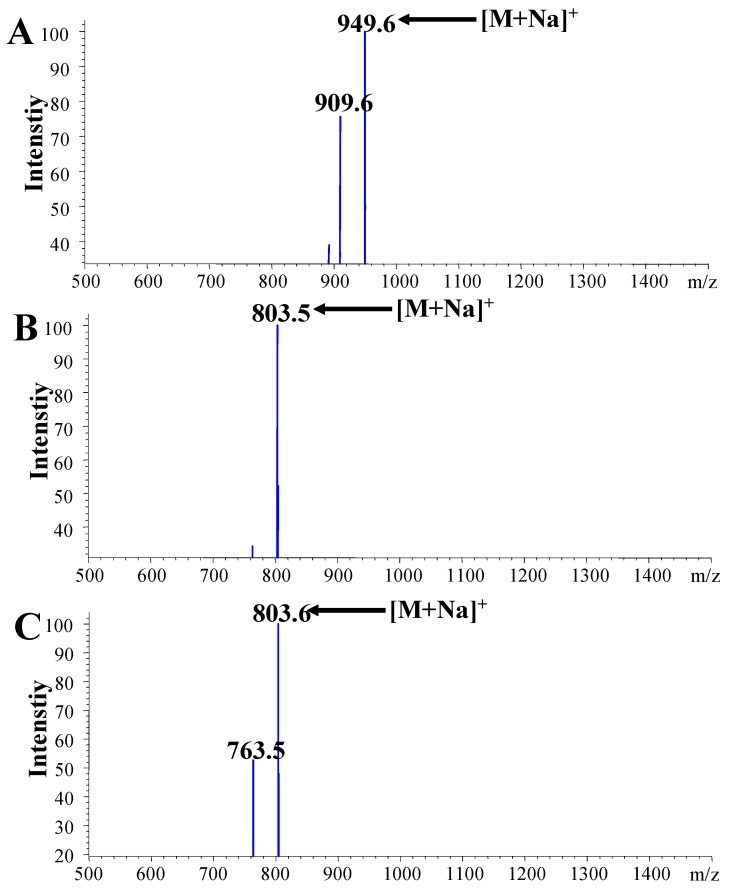
ESI/MS (+) spectra of saikosaponin c (A); saikosaponin a (B); saikosaponin d (C).

In negative ion mode, strong [M − H]^−^ and [M − H + HCOOH + H_2_O]^−^ signals of saikosaponin a as well as d (MW − 780) were observed at *m/z* 779.4 and 842.4, respectively. However, only the [M − H + HCOOH + H_2_O]^−^ signal of saikosaponin c (MW − S926) at *m/z* 988.5 is shown in Figure 4. Although protonated adducts of these saikosaponins were not observed in positive ion mode, saikosaponins c, a, and d gave obvious sodium adduct peaks at *m/z* 949.6, 803.5 and 803.6, respectively (Figure 5). Comparing the negative and positive ion modes, the former is suitable for detection and quantitation of three saikosaponins in *B. falcatum*. Finally, the identification of these saikosaponins was achieved by comparison to authentic reference substance and their retention time sequence [19].

### 2.4. Linearity and Calibration Curves

As shown in Table 1, the peak area ratios of three saikosaponins in real samples varied linearly with concentration over the range 0.75–15.0 mg/mL. Then calibration model was selected based on the analysis of data by linear regression with intercept (*y* = a*x* + b). Moreover, the regression coefficient (*R*^2^) of three saikosaponins were greater than 0.9995.

### 2.5. Limit of Detection (LOD) and Quantification (LOQ)

LOD and LOQ of three saikosaponins under the present chromatographic conditions were determined on the basis of response and slope of each regression equation at a signal-to-noise (S/N) of 3 and 10. They ranged in 32.4–66.5 μg/mL and 105.6–220.2 μg/mL, respectively. The results are summarized in Table 1.

### 2.6. Precision

The quality control samples at low, medium and high were analyzed in a set of five on a single assay day to determine intra-day precision, and analyzed in duplicated on each of three consecutive days for inter-day variation. The values of relative standard deviations (R.S.D.s) of the intra-day and inter-day measurement variations were all less than 2.8% for three saikosaponins (*n* = 5).

### 2.7. Repeatability

In order to test the repeatability, six sample solutions of *B. falcatum* were prepared. The contents of three saikosaponins were 2.11, 3.05 and 3.35 mg/g, and R.S.D.s. were 1.13%, 1.25 % and 1.25%, respectively. Thus repeatability was very good.

### 2.8. Stability

In the stability test, the dry residue samples stored at −20 °C after extraction were found to be stable for 12 days with R.S.D.s value ≤4.5% at all eight concentration levels. Furthermore, the same sample solution was analyzed every 24 h over 6 days at the room temperature. The R.S.D.s. of contents of the three saikosaponins in the same sample ranged between 1.35% and 2.03%, which indicated that the sample was stable over 6 days under the experimental conditions.

### 2.9. Recovery

In order to evaluate the accuracy of this method, recovery was performed by adding standard solutions at low, medium and high levels (50%, 100 % and 150%) to 1.0 g *B. falcatum* roots with known content of three saikosaponins. The recoveries of analytes were calculated as follows:

$$\%\;\mathrm{Recovery}\;=\frac{\mathrm{Observed}\;\mathrm{concentration}}{\mathrm{Spiked}\;\mathrm{concentration}}\times 100$$

The samples (*n* = 3) were then extracted according to the procedure described above and analyzed. The recovery of each saikosaponin was calculated as the percentage of the net amount of each saikosaponins obtained after extraction from that had been added prior to the extraction, the recovery results were summarized in Table 2. It was indicated that the extraction method was efficient enough of determination of the three saikosaponins in *B. falcatum*.

### 2.10. Application of the Method to Real Sample

The established LC-DAD-ESI/MS method was applied to determination in triplicate of three saikosaponins in *B. falcatum*. The total contents (*n* = 3) of three saikosaponins was up to 8.51 ± 0.23 mg/g.

## 3. Experimental

### 3.1. Reagents and Materials

*B. falcatum* roots were provided and identified by Wei Li (College of Chinese Material Medicine, Jilin Agricultural University). Standards of saikosaponins c, a, and d were obtained from National Institute for the Control of Pharmaceutical and Biological Products of China. Acetonitrile was of HPLC grade from Fisher Chemicals (USA). Other chemicals, such as methanol, ethanol and formic acid, were all of analytical grade from Beijing Chemical Factory. Water was purified using a Milli-Q water purification system (Milipore, USA).

### 3.2. LC-DAD-ESI/MS Conditions

In present work, three saikosaponins in *B. falcatum* were quantified by high performance liquid chromatography coupled with diode-array detector (HPLC-DAD). The HPLC analyses were performed with a HPLC instrument (Agilent 1100, USA) equipped with a binary pump, on-line degasser, autosampler and a column oven. Separation was achieved on a ZORBAX SB-Aq column (150 mm × 4.6 mm, 5 μm) from Agilent Analytical Instruments Co., Ltd. The column temperature was set at 30 °C and detection wavelength was set 204 nm. The mobile phase was consisted of acetonitrile (A) and water (B) containing 0.5% formic acid with flow rate of 0.8 mL/min. The gradient elution was programmed as follows: 0–30min, 30–70% A. The sample solution (20 μL) was directly injected into the chromatographic column. The chromatographic peaks of three saikosaponins were confirmed by comparing their retention time with those of the reference standards. Quantification was carried out by the integration of the peak using external standard method.

The HPLC-DAD system was interfaced to the MS detector. An electrospray ionization source was used in positive and negative ion mode [ESI]. The capillary potential was 4.0 kV, the dry gas temperature 350 °C, the drying gas flow 10 L/min, and the ionizing voltage of 40 eV. Total ion chromatograms from *m/z* 500 to 1,500 were obtained. (Figure 2B–C). 

### 3.3. Preparation of Standard Solutions

Standards of the three saikosaponins dissolved in mobile phase (30% acetonitrile) were prepared from stock standard solutions containing 7.5 mg/mL of saikosaponin c, 15.0 mg/mL of saikosaponin a, and 10.8 mg/mL of saikosaponin d. The stock solutions were serially diluted, mixed and used for preparation of standard solutions, which were stored at 4 °C. Calibration curves were established based on eight concentrations with the ranges of 0.75–7.5 mg/mL for saikosaponin c, 1.5–15.0 mg/mL for saikosaponin a and 1.08–10.8 mg/mL for saikosaponin d by diluting these stocking solution in series.

### 3.4. Sample Preparation

The dried roots of *B. falcatum* were ground to a powder and sieved through a 40-mesh screen. The powder was dried at 60 °C until constant weight and was well blended before use. An ASE 100 System (Dionex, Sunnyvale, CA, USA) with 34 mL stainless steel ASE vessels was used for the pressurized liquid extraction. Extraction conditions were optimized by single-factor experiments (sequentially varying the experimental parameters, one at a time, while all the other parameters remained fixed). The extract was evaporated to dryness using a rotary evaporator under 45 °C. The residue was then dissolved in 5.0 mL of methanol and filtered through a 0.45 μm nylon filter membrane prior to injection into the LC-DAD-ESI/MS system.

### 3.5. Extraction Yield Determination

The saikosaponins yield is calculated as following formula:

$$\mathrm{Yield}\;(\mathrm{mg}/g)=\frac{\mathrm{weight}\;\mathrm{of}\;\mathrm{saikosaponins}\;\mathrm{extracted}\;(\mathrm{mg})}{\mathrm{weight}\;\mathrm{of}\;\mathrm{dried}\;\mathrm{sample}\;(g)}$$

## 4. Conclusions

The proposed LC-DAD-ESI/MS method has been applied successfully to the simultaneous determination of three saikosaponins in *B. falcatum*. Additionally, the method was validated for good linearity, limit of detection, limit of quantitation, precision, repeatability, stability and recovery. This simple, rapid, low-cost and reliable LC-DAD-ESI/MS method is therefore suitable for routine quantitative analysis and quality control of *B. falcatum* containing bioactive multi-components.

## Figures and Tables

**Table 1:** Linear regression equation, correlation coefficient and linearity of three saikosaponins in *B. falcatum*.

Compound	Regression equation	*R*^2^	Linearity range (mg/mL)	LOD (μg/mL)	LOQ (μg/mL)
Sc	y = 2149.2 x − 91.46	0.9997	0.75–7.5	32.4	105.6
Sa	y = 4252.8 x − 60.97	0.9995	1.5–15.0	66.5	220.2
Sd	y = 3929.7 x − 277.71	0.9996	1.08–10.8	45.2	145.6

Sc, saikosaponin c; Sa, saikosaponin a; Sd, saikosaponin d; LOD, limits of detection; LOQ, limits of quantification.

**Table 2:** Recovery test of the three saikosaponins in *B. falcatum* (*n* = 3).

Components	Spiked (mg/mL)	Original (mg/mL)	found (mg/mL)	Mean Recovery (%)	R.S.D.(%) (*n* = 3)
Sc	1.06	2.11	3.21	101.5	2.2
	2.11	3.17	4.20	99.6	1.6
	3.17	4.22	5.55	105.2	1.3
Sa	1.53	5.28	4.88	106.6	2.1
	3.05	4.58	6.46	105.9	1.7
	4.58	6.10	8.03	105.3	1.5
Sd	1.68	7.63	4.93	98.1	2.8
	3.35	5.03	7.12	106.2	1.2
	5.03	6.70	8.09	96.6	1.4

Sc, saikosaponin c; Sa, saikosaponin a; Sd, saikosaponin d.
